# Stereotactic body radiotherapy based treatment for hepatocellular carcinoma with extensive portal vein tumor thrombosis

**DOI:** 10.1186/s13014-018-1136-5

**Published:** 2018-09-25

**Authors:** Yongjie Shui, Wei Yu, Xiaoqiu Ren, Yinglu Guo, Jing Xu, Tao Ma, Bicheng Zhang, Jianjun Wu, Qinghai Li, Qiongge Hu, Li Shen, Xueli Bai, Tingbo Liang, Qichun Wei

**Affiliations:** 1 Department of Radiation Oncology, The Second Affiliated Hospital, Zhejiang University School of Medicine, Jiefang Road 88, Hangzhou, 310009 People’s Republic of China; 2 Department of Hepatobiliary and Pancreatic Surgery, Zhejiang Provincial Key Laboratory of Pancreatic Disease, The Second Affiliated Hospital, Zhejiang University School of Medicine, Hangzhou, 310009 People’s Republic of China; 3 Department of Radiology, The Second Affiliated Hospital, Zhejiang University School of Medicine, Hangzhou, 310009 People’s Republic of China; 4 Ministry of Education Key Laboratory of Cancer Prevention and Intervention, Zhejiang University Cancer Institute, Hangzhou, 310009 People’s Republic of China

**Keywords:** Hepatocellular carcinoma, Portal vein tumor thrombosis, Stereotactic body radiotherapy, Transarterial chemoembolization

## Abstract

**Background:**

There is currently no worldwide consensus for the management of hepatocellular carcinoma (HCC) with portal vein tumor thrombus (PVTT). We evaluated the efficacy of stereotactic body radiotherapy (SBRT) as the initial treatment for HCC with extensive PVTT based on a relatively large number of patients.

**Methods:**

In our multidisciplinary approach for patients with hepatobiliary tumors, SBRT is recommended for unresectable HCC with PVTT or those with contraindication for transarterial chemoembolization (TACE). The aim is to shrink the tumor thrombus and preserve adequate portal venous flow, thus facilitating subsequent treatments such as TACE and tumor resection. In the present study, 70 continuous cases of HCC patients with extensive PVTT initially treated with SBRT were studied. The median follow-up period was 9.5 months (range, 1.0–21.0 months). The dynamic changes of tumor thrombosis with time after SBRT were also analyzed.

**Results:**

The median survival time for the whole group was 10.0 months (95% CI, 7.7–12.3 months), with a 6- and 12-month overall survival (OS) rate of 67.3%, and 40.0% respectively. Patients who received combined SBRT and TACE showed significantly longer OS than those without indication for TACE after SBRT (12.0 ± 1.6 vs. 3.0 ± 1.0 months). Patients with good response to radiation usually had better survival. SBRT was well tolerated in our patient series.

**Conclusions:**

In conclusion, SBRT used as the initial treatment for HCC patients with extensive PVTT originally unsuitable for resection or TACE can achieve adequate thrombus shrinkage and portal vein flow restoration in the majority of cases. It could thus offer the patients an opportunity to undergo further treatment such as resection or TACE procedure. Such therapeutic strategy may result in survival advantage, especially for those who do receive combined modality with SBRT.

## Background

Hepatocellular carcinoma (HCC) is the sixth most prevalent cancer worldwide [1]. In China, HCC is the fourth most commonly diagnosed cancer and the third leading cause of cancer death [2]. Macrovascular invasion (MVI) is common in HCC; in such case, tumor cells invade the portal veins, hepatic veins, or the inferior vena cava in the liver [3, 4]. Portal vein tumor thrombus (PVTT) is the most common form of MVI in HCC, with an incidence ranging from 44 to 62.2% [5]. About 10% to 60% of HCC patients have PVTT at the time of diagnosis [6, 7]. Although the survival rate of patients with HCC has been improved recently, the prognosis for those with PVTT remains poor, as their median survival is only 2–4 months via supportive care [8]. Overall, PVTT plays a major role in predicting the therapeutic outcome and clinical staging of HCC [9, 10].

There is currently no widely-accepted consensus for the management of HCC with PVTT. According to some guidelines in Europe and America, HCC with PVTT is regarded as Stage C per Barcelona Clinic Liver Cancer (BCLC) Staging system, and sorafenib alone is recommended as the treatment of choice [11]. In Southeast Asian countries, modalities including surgery, radiotherapy, transarterial chemoembolization (TACE), and/or sorafenib are involved in the multidisciplinary treatment of HCC with PVTT [12, 13]. Surgical treatment is recommended for suitable HCC patients with type I/II PVTT [14–16]. However, the management of HCC with extensive portal vein involvement remains complicated and controversial. Patients with PVTT extended to the main portal vein or the contralateral branch had no survival benefit after surgical treatment [17]. TACE has been contraindicated in the treatment of HCC patients with PVTT involving the main trunk and/or first branch of the portal vein due to potential risk of liver infarction and hepatic failure resulting from ischemia [6, 18]. Several recent reports have shown that selected patients with PVTT may benefit from more aggressive treatment modalities [6, 19] such as a combination of radiotherapy and TACE. So far, few studies have investigated the efficacy of stereotactic body radiotherapy (SBRT) for the treatment of PVTT [20–22].

SBRT has emerged as a new technology which delivers large dose of radiation to a target in a few fractions. By taking advantage of the technologic advancements in precise radiation dose delivery, respiratory motion management, and daily image guidance, SBRT enables accurate targeting of multiple high-dose radiation beams to treat a tumor volume, typically over 1 to 5 fractions. Characterized by rendering a higher biologically effective dose (BED) than conventionally fractionated radiotherapy, minimal invasiveness and decreased morbidity, SBRT can be finished in a week. Such relatively short treatment course may post less interference with other therapeutic measures which may also benefit the patient sequentially in time.

In our multidisciplinary management for patients with hepatobiliary tumors, SBRT is recommended for patients with unresectable HCC with PVTT and those with contraindication for TACE. The aim is to shrink the tumor thrombus and preserve adequate portal venous flow, thus facilitating subsequent treatments such as TACE or tumor resection. In the present report, 70 continuous HCC with extensive PVTT initially treated with SBRT were analyzed. The therapeutic response, survival, safety, and treatment strategy were discussed.

## Methods

### Patient population and radiation treatment

**Fig. 1:**
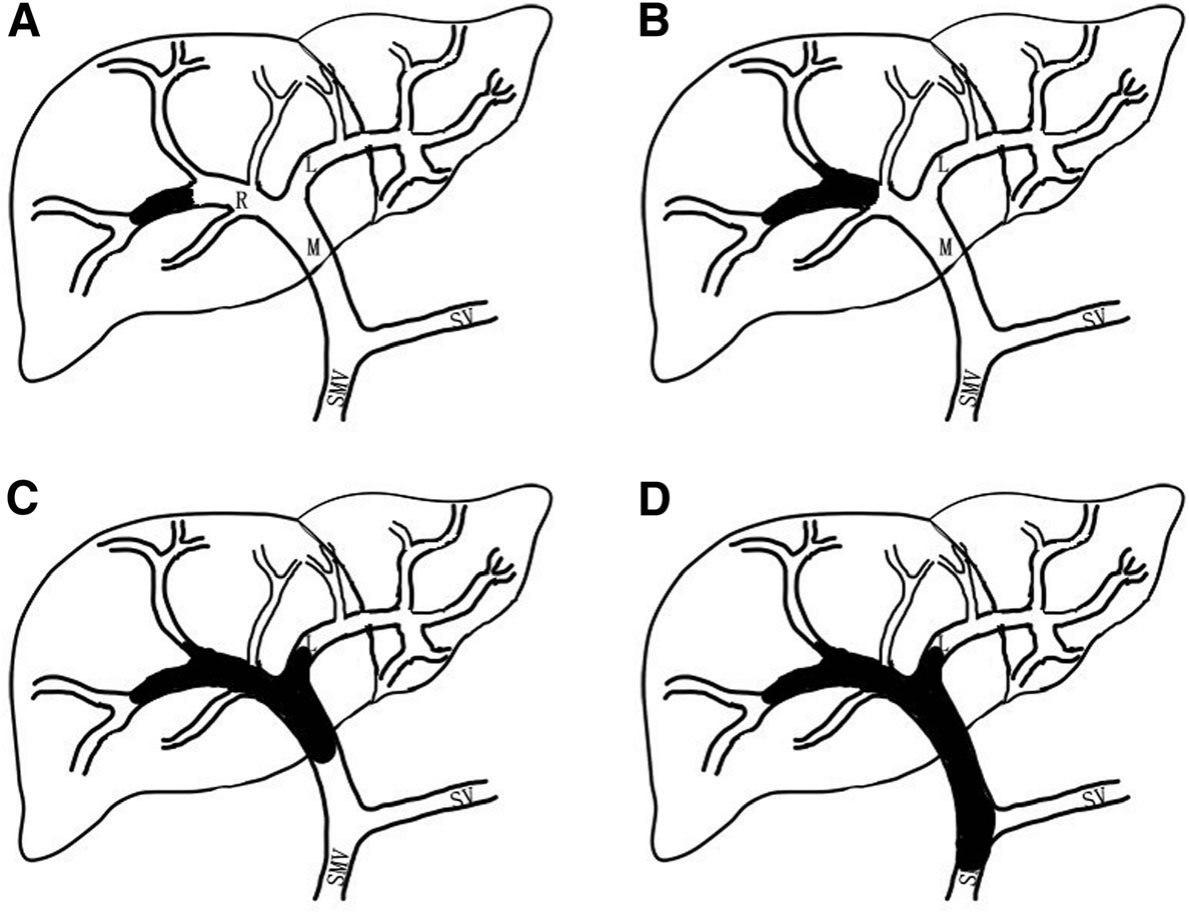
Cheng’s classification of hepatocellular carcinoma with portal vein tumor thrombus. **a** type I, tumor thrombus involving segmental or sectoral branches of the portal vein or above; **b** type II, tumor thrombus involving the right/left portal vein; **c** type III, tumor thrombus involving the main portal vein; **d** type IV, tumor thrombus involving the superior mesenteric vein

This retrospective study was performed with the approval of our local ethics committee. From December 2015 to June 2017, 70 continuous HCC patients with PVTT received SBRT at the Second Affiliated Hospital, Zhejiang University School of Medicine. The diagnosis of HCC was based on the American Association for the Study of Liver Disease (AASLD) guideline [23]. Portal vein invasion was identified by the presence of a low-attenuation intraluminal filling defect adjacent to the primary tumor as discerned from contrast-enhanced computed tomography (CT). Cheng’s classification of PVTT was applied in this study, and comprised of four levels based on the extent of tumor thrombus in the portal vein (Fig. 1): type I_0_, tumor thrombus found only under microscopy; type I, tumor thrombus involving segmental or sectoral branches of the portal vein or above; type II, tumor thrombus involving the right/left portal vein; type III, tumor thrombus involving the main portal vein; and type IV, tumor thrombus involving the superior mesenteric vein [24]. Basic criteria for SBRT: (1) tumor thrombus involving the main trunk and/or first branches of the portal vein, unsuitable for surgery or TACE; (2) an Eastern Cooperative Oncology Group (ECOG) performance status (PS) of 0–2; (3) no refractory ascites; (4) Child–Pugh class A and B, or class C with good performance status; (5) no previous radiotherapy to the liver; and (6) more than 700 cc of uninvolved liver. A preliminary estimate of the uninvolved liver volume was made using 3D imaging software (IQQA-Liver, EDDA Technology Inc., Princeton, NJ, USA), whereas its exact volume was verified from the subsequent SBRT planning dosimetry (Eclipse software, Varian® Medical Systems, Palo Alto, CA).

All patients were immobilized in a stereotactic body frame (Karity, Guangzhou, China) with customized vacuum cushion and abdominal compression for control of respiratory motion. Oral contrast agent (50 ml of 3% Ioversol) was administered before CT simulation. Four-dimensional contrast-enhanced computed tomography (4DCT) simulation (Light Speed RT, GE) was performed at 2.5-mm slice thickness. For breathing movement amplitude too small to be detected during 4DCT scanning, scans were taken at end-expiration phase, end-inspiration phase, and during quiet free-breathing under abdominal compression. The CT scan during free breathing was then used for treatment planning. The gross tumor volume (GTV) represented the tumor thrombosis visualized on the contrast enhanced CT, and magnetic resonance (MR) images. If the primary hepatic lesion was small (less than 5 cm) and adjacent to the PVTT, both portal vein tumor thrombus and the primary lesion were contoured as the GTV. Internal target volume (ITV) was defined as the volumetric sum of GTVs in the multiple phases. The planning target volume (PTV) included ITV with 3–5 mm margins, to account for daily set-up variations. PTV was adjusted manually to minimize overlapping the gastrointestinal tract when indicated. The mean volume of PTV was 390.8 ± 37.6 cm^3^, varying widely from a minimum of 63.0 cm^3^ to a maximum of 1452.9 cm^3^. Plans were devised such that the prescription dose was prescribed at the isodose line encompassing > 95% of the PTV. The preferred maximum dose within the PTV was between 110 and 130% of the prescribed dose. The median prescription dose to PTV was 40 Gy (range, 25–50) in five fractions administered over a week. Organs at risk (OARs) included liver, kidneys, stomach, duodenum, small intestine, colon and spinal cord. Dose-volume planning objectives for the OARs were defined as follows (Table 1): normal liver, mean dose ≤15 Gy; bilateral kidney, mean dose ≤12 Gy; and spinal cord, maximal dose<27 Gy. The maximal dose to 1 cc (D1cc) was limited to 31 Gy for the gastrointestinal tract including stomach, duodenum, small intestine and colon. Normal liver volume was defined as the total liver volume minus the PTV. Target and OAR contouring were performed using Varian Dosimetrist and Oncologist software (Eclipse software, Varian® Medical Systems, Palo Alto, CA). Treatment was delivered with a Varian® Trilogy™ linear accelerator (Varian® Medical Systems, Palo Alto, CA) using X-ray beams of 6–10 MV energy. Daily image guidance was performed by means of kilovoltage cone-beam CT, with patients’ 3D positioning verified prior to each radiation treatment session.

**Table 1:** Dose-volume constraints to organs at risk

Organs at risk	Constraints (5 Fractions)
Liver	mean dose ≤15 Gy, >700 cc
Kidney	mean dose ≤12 Gy V18 < 33%
Spinal cord	maximal dose < 27 Gy
Stomach	V31 < 1 cc V20 < 3 cc
Duodenum	V31 < 1 cc V20 < 3 cc
Small intestine	V31 < 1 cc V20 < 3 cc
Colon	V31 < 1 cc V20 < 10 cc

Abbreviations: Vxx = the volume or percentage of organ receiving more than the xx Gy

### Evaluation

Patients were assessed weekly for toxicities in the first month after SBRT, monthly for the following two months, and once every three months thereafter. Treatment-associated acute and late toxicities were scored according to the Common Terminology Criteria for Adverse Events (CTCAE; version 3.0). Tumor response was assessed using the modified Response Evaluation Criteria in Solid Tumors (mRECIST) criteria [25]. The response of PVTT to SBRT was evaluated by dynamic contrast enhanced CT and/or MRI at 1, 3, and then every 3 months after SBRT. Biochemical response was assessed in patients with elevated alpha-fetoprotein (AFP) level before radiotherapy and defined as either a ≥ 50% reduction or normalization of the AFP level within one month after SBRT.

### Follow-up and statistical analysis

The cutoff date for the last follow-up was February 28, 2018, for the censored data analysis. The median follow-up period was 9.5 months (range: 1.0–21.0). The overall survival (OS) was calculated from the start of SBRT to the date of either death or the last follow-up visit. The Kaplan-Meier method was used to analyze the OS, with log-rank test used to examine group differences. Cox regression model was used for multivariate analysis. All statistical analyses were performed using SPSS software package (version 20.0; SPSS Inc., Chicago). A *p*-value of <0.05 was considered statistically significant.

## Results

A total of 70 HCC patients with PVTT were irradiated and included in this analysis. Table 2 shows the patients’ characteristics, classification of PVTT and radiotherapy scheme. The median age at diagnosis of PVTT was 53.8 years (range: 25–75). The median time from primary HCC diagnosis to SBRT treatment was 7 months (range: 0–85). The median time interval between diagnosis of PVTT and SBRT treatment was 1.2 months (range 0–12). Tumor thrombosis involving the first order portal vein branches without main portal vein involvement (TypeII) was found in 42 patients (60%). Twenty- seven patients (38.6%) had tumor thrombosis invading the main trunk (Type III), one patient (1.4%) had tumor thrombosis invaded the superior mesenteric vein, portal vein main trunk and both first branches (Type IV).

**Table 2:** Patient characteristics

Characteristics	n (%)
Age, y	
≥ 50	48 (68.6)
< 50	22 (31.4)
Gender	
Male	59 (84.3)
Female	11 (15.7)
Therapeutic modalities	
SBRT alone	20 (28.6)
SBRT+TACE	46 (65.7)
SBRT+Surgery	4 (5.7)
Dose, Gy	
≤ 35.0	29 (41.4)
≥ 40.0	41 (58.6)
Types of PVTT	
II	42 (60.0)
III	27 (38.6)
IV	1 (1.4)
HBsAg	
Negative	12 (17.1)
Positive	58 (82.9)
Child-Pugh classification	
A	45 (64.3)
B	24 (34.3)
C	1 (1.4)
PS (ECOG)	
0	56 (80.0)
1	14 (20.0)
Origination of PVTT	
Right branch	49 (70.0)
Left branch	21 (30.0)
AFP, ng/L	
≤ 20	13 (18.6)
21 ~ 399	17 (24.3)
≥ 400	40 (57.1)
PLT, 10^9^/L	
≥ 100	39 (55.7)
< 100	31 (44.3)
HGB, g/L	
≥ 120	42 (60.0)
< 120	28 (40.0)
TBIL, μmol/L	
≥ 20	34 (48.6)
< 20	36 (51.4)
Albumin, g/L	
≥ 35	41 (58.6)
< 35	29 (41.4)
ALT, U/L	
≥ 50	25 (35.7)
< 50	45 (64.3)
AST, U/L	
≥ 50	48 (68.6)
< 50	22 (31.4)

Abbreviations: *SBRT* Stereotactic body radiotherapy, *Types* types of tumor thrombi, *HBsAg* Hepatitis B surface antigen, *PS* Performance status, *ECOG* Eastern Cooperative Oncology Group, *AFP* Alpha–fetoprotein, *PLT* Platelet, *HGB* Hemoglobin, *TBIL* Total bilirubin, *ALT* Alanine aminotransferase, *AST* Aspartate aminotransferase

Twenty patients (28.6%) received SBRT alone. Another 46 (65.7%) patients received TACE (1–5 cycles, median 2.4 cycles) after SBRT; among them, 3 underwent further radioactive seeds implantation, and one received radiofrequency ablation (RFA) after TACE. The final 4 (5.7%) patients underwent surgery, including 3 with respective liver lobectomy at 3, 8 and 10 weeks after SBRT, and one patient underwent liver transplantation 4 weeks after SBRT.

### PVTT response

Fifty-three cases had contrast-enhanced CT/MRI around 4 weeks after SBRT, partial response (PR), stable disease (SD), and progression disease (PD) were observed in 41 (77.4%), 8 (15.1%), and 4 (7.5%), respectively. While none had achieved complete response (CR) by this time point, there were 5 patients (9.4%) reached near CR with minimal residual disease less than 10% of original enhanced size. Local control (LC) (inclusive of partial and stable response) was thus achieved in 92.5% of the treated lesions.

At 3 months after SBRT, 62 patients were assessed for PVTT therapeutic responses. CR, PR, and SD were observed in 6 (9.7%), 43 (69.4%), and 4 (6.4%) of the patients, respectively. LC (inclusive of CR, PR and SD) was achieved in 85.5% of the treated lesions. The rest 9 patients (14.5%) had PD, including 3 cases having developed PD at the first follow-up.

Six months after SBRT, assessments of PVTT therapeutic response were done in 31 patients, CR, PR, SD, and PD were observed in 10 (32.2%), 16 (51.6%), 2 (6.4%), and 3 (9.8%) (including 1 case evaluated as PD beforehand) patients, respectively. Sixteen patients had follow-up imaging around 9 months after SBRT, and the respective CR and PR rate were 56.25% (9 patients) and 43.75% (7), with the corresponding figures at 12 months being 66.7% (10) and 33.3% (5).

Of the 57 patients with elevated AFP levels before SBRT, 29 patients (54.7%) exhibited ≥50% reduction in the AFP levels within one month after SBRT, with 6 cases (11.3%) had AFP in the normal range.

### Overall survival

**Fig. 2:**
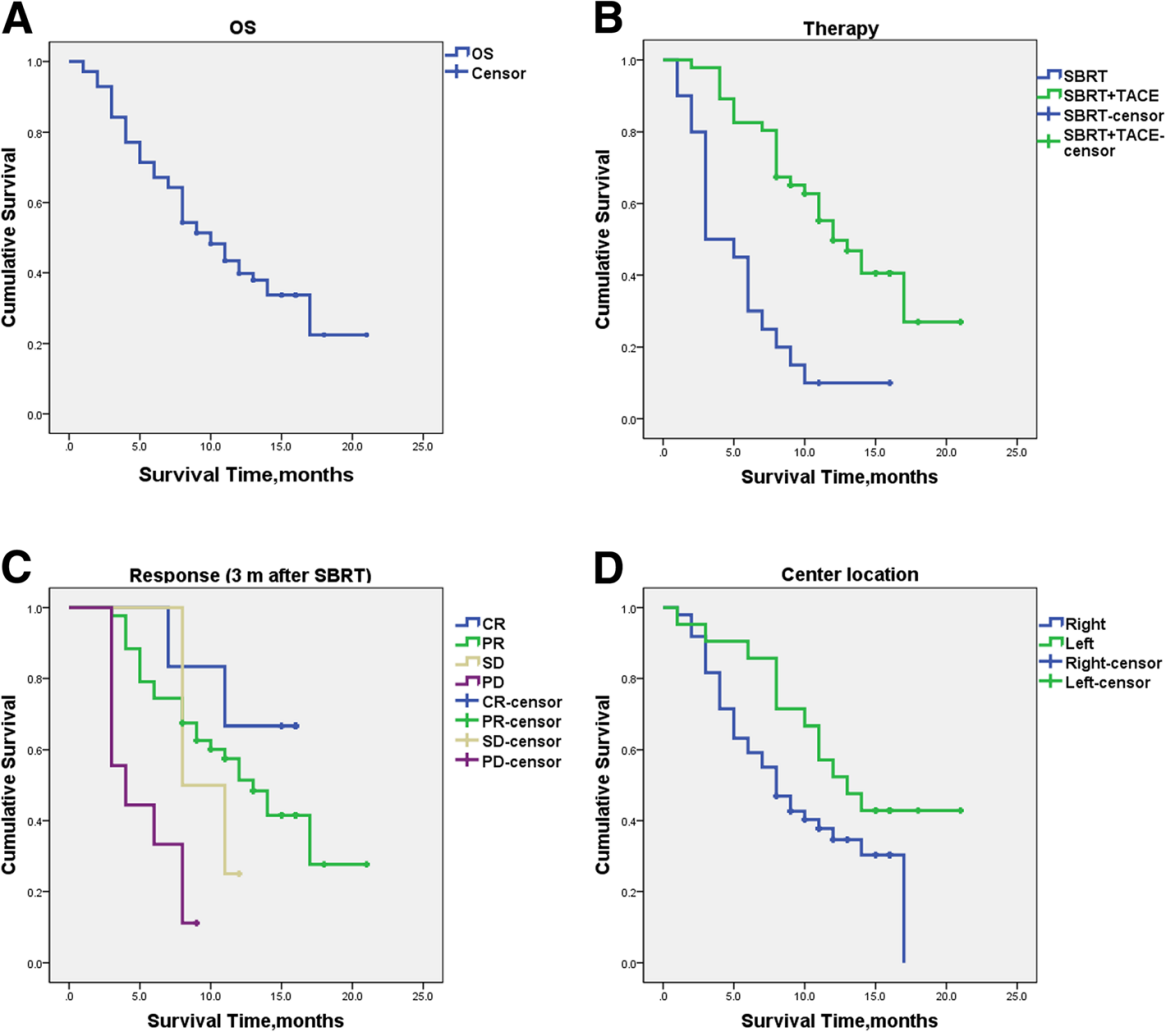
Overall survival of the whole group and subgroup. **a** Overall survival curve for the whole group of 70 HCC patients with PVTT; **b** Overall survival curves according to the additional therapy; **c** Overall survival curves according to the response at three months after completion of SBRT; **d** Overall survival curves according to the center location of PVTT

With median follow-up period of 9.5 months, 25 patients (35.7%) were still alive at the time of the current analysis. The median survival time for the whole group was 10.0 months (95% CI, 7.7–12.3), with 6- and 12-month OS rates of 67.3%, and 40.0%, respectively (Fig. 2a).

Median survival times were 12.0 ± 1.6 and 3 ± 1.0 months for those receiving TACE after SBRT and SBRT alone, respectively. Patients who received combined treatments of SBRT and TACE showed significantly longer OS than those unable to undergo TACE after SBRT (*p* < 0.001; Fig. 2b). The 6- and 12-month OS rates were 82.8%, and 49.7% for patients receiving SBRT plus TACE, and 30.0%, and 10.0% for those receiving SBRT alone. Four patients underwent tumor resection after SBRT, with 1 patient died at the fourth month while the other 3 were alive at their respective follow-up time of 13, 15 and 15 months.

The median survival time for patients with PR, SD and PD of the PVTT at 3 months after completion of SBRT was 13.0, 8.0 and 4.0 months respectively. The median survival for the 6 patients with CR was deficiency because only 2 patients died at seventh and eleventh month. The other 4 patients remained alive, with 1 patient assessed at 15-month and 3 at 16-month follow-up points after SBRT. The median survival times for patients with LC (inclusive of CR, PR and SD) of the PVTT were better than those with PD after SBRT (13.0 vs. 4.0 months, *p* < 0.001) (Fig. 2c).

Interestingly, patients with PVTT at the left branch location seemed to have longer median OS than those with thrombosis in the right branch of portal vein (13.0 vs. 8.0 months, *P* = 0.079) (Fig. 2d, Table 3).

**Table 3:** Analysis of prognostic factors for survival

	N	Median survival (month; 95% CI)	*p* values
Gender			0.13
Male	59	11.0 (7.6–14.4)	
Female	11	8.0 (1.5–14.5)	
Age, y			0.033
<50	22	17.0 (17.0–17.0)	
≥ 50	48	8.0 (6.3–9.7)	
AFP, ng/L			0.438
≤ 20	14	8.0 (2.9–13.1)	
21–399	18	14.0 (11.9–16.1)	
≥ 400	38	8.0 (5.6–10.4)	
Additional treatment after RT			0.00
SBRT alone	20	3.0 (1.1–4.9)	
SBRT+TACE	46	12.0 (8.9–15.1)	
Radiation Dose			0.376
≤ 27.5Gy	4	3.0 (0–8.9)	
30-35Gy	25	8.0 (6.8–9.2)	
≥ 40Gy	41	11.0 (7.7–14.3)	
Origination of PVTT			0.079
Right branch	49	8.0 (5.8–10.2)	
Left branch	21	13.0 (8.5–17.5)	
Type of tumor thrombosis			0.107
Type II	42	8.0 (5.5–10.5)	
Type III	27	14.0 (8.6–19.4)	
Response (3 m after SBRT)			0.000
CR	6	*	
PR	43	13.0 (10.0–16.0)	
SD	4	**	
PD	9	4.0 (1.1–6.9)	

Abbreviations: *SBRT* Stereotactic body radiotherapy, *AFP* Alpha-fetoprotein, *RT* Radiotherapy, *CR* Complete response, *PR* Partial response, *SD* Stable disease, *PD* Progressive disease. * The medium survival for patients with CR was not reported because only two patients died in 7th and 11th month. The other four patients were alive, 1 patient with 15 months follow-up, 3 patients with 16 months follow-up after SBRT. ** In the four patients with SD, 3 patients died in the 8th, 8th and 11th month, the other patient was alive with 12 months follow-up

### Toxicity

Patients tolerated the SBRT generally well, since a very mild pattern of acute toxicity was observed. No treatment-related deaths or serious adverse events were seen within 3 months after SBRT. Acute radiation side effects were mild, including nausea, vomiting, anorexia, and abdominal pain in some cases. Grade-3 leukopenia and thrombocytopenia were seen in 5 (7.1%) and 4 (5.7%) patients, respectively. Grade-3 liver enzyme and bilirubin elevation were found in 3 (4.3%) and 8 (8.6%) cases, while grade-3 albumin decrease was seen in 11 (15.7%). No grade-4 hematologic toxicity, liver enzyme, bilirubin and albumin level change was seen. No radiation induced liver disease was encountered in the entire patient group. Later toxicities such as gastrointestinal stenosis, bleeding, perforation and ulcer were not observed during the follow-up periods.

Only one patient (1.4%) showed progression of Child-Pugh class from A to C within 3 months after SBRT, while progression from class B to C was observed in 2 cases. All these 3 patients were deteriorated due to intrahepatic tumor progression. Downgrading of Child-Pugh class from B to A was observed in 3 cases.

## Discussion

In the present study, SBRT was used as the first therapy for the management of HCC patients with extensive PVTT. These patients were originally unsuitable for surgery or had contraindication for TACE. SBRT induced prominent response in the PVTT. The objective response rate at 1, 3, 6 months were 77.4%, 79.1% and 83.8%, respectively. Within 1 month after SBRT, 5 cases reached near CR, i.e., minimal residual disease less than 10% of initial lesion. Remarkably, the CR rate increased with time, from 9.7% at 3 months to 32.2% at 6 months after SBRT. For the 16 patients with imaging reassessment at 9 months after treatment, more than half achieved CR. Progression after SBRT was found in about 17.1% of the cases, with most PD occuring within the first 3 months. In our series, 4 patients had PD of the PVTT within 1 month, 6 between 1 and 3 months, and only 2 patients at 6 months. To the best of our knowledge, this appears to be the first report describing the dynamic changes of tumor thrombosis after SBRT.

Around 70% of our PVTT cases shrank to a degree of PR in the first few weeks. Most achieved restoration of portal vein flow, which relieved the associated symptoms and made it feasible for patients to endure further interventional therapies. The strategy seems practical to shrink the thrombosis and restore the portal flow by SBRT, thus enable the patients to have the opportunity to receive subsequent treatments, such as tumor resection or TACE procedure.

The median survival time from the start of SBRT for this patient series was 10.0 months, with the respective 6- and 12-month OS rates of 67.3% and 40.0%. Published results concerning SBRT in the treatment of PVTT are scarce, and median survival time has been reported to vary from 8 to 13 months, with 1-year OS rate from 43.2 to 50.3% [22, 26, 27]. In a prospective study of phase I and II trials reported by Bujold et al. [28], 56 HCC patients with tumor thrombosis showed a 1-year OS rate of 44% after SBRT, with which our result seems comparable. With a median survival time of 12 months, our patients who got the chance to receive TACE after SBRT had longer survival time that is comparable to the results of the previous reports [27]. It is remarkable that patients in the present study were originally considered to harbor unresectable tumor or had contraindication for TACE. This subgroup of patients was expected to have lower OS rate. In view of such adverse baseline characteristics, the therapeutic outcome with median OS of 10 months seems satisfactory.

For the 5 patients who achieved near CR within the first few weeks, all were alive at the last follow-up times of 20, 17, 17, 16, and 10 months respectively. Those had CR at 3 months after SBRT also showed longer OS. Patients with good PVTT response to SBRT usually have better survival. As for the radiation dose, patients receiving 40 Gy or more seems to have longer OS (Table 3). Hence, We suggest higher radiation dose (≥40Gy in 5 fractions) for the SBRT treatment of PVTT, with the aim of achieving adequate therapeutic response and better survival.

The natural history of HCC patients with PVTT dictates only 2.4–4 months of survival duration [8]. Although Sorafenib provided a significant survival benefit in randomized phase III trials, the gain was modest (2–3 months) [29, 30]. Radiotherapy may improve survival chance for such patients. Yeh et al. reported a large series of HCC with PVTT treated by conventional three-dimensional conformal radiotherapy (3D-CRT), but the median survival time was only 7.0 months [31]. Matsuo et al. compared the efficacy of SBRT with 3D-CRT in the management of PVTT and better therapeutic results for the former was observed, with 1-year OS rates of 49.3% in SBRT group vs. 29.3% in 3D-CRT group. Taking all the results into consideration, SBRT seems to be of therapeutic benefit for HCC patients with PVTT, especially for those who had contraindication for TACE or surgery.

SBRT was well tolerated in our patient series. Only mild radiation acute side effects were observed in some of cases. No treatment-related deaths or serious adverse events were encountered. Furthermore, no radiation induced liver disease was observed, even in those receiving TACE after SBRT. Those who experienced deterioration of liver function were mainly due to intrahepatic tumor progression. The low toxicity of SBRT in the management of PVTT was in line with the results of other SBRT studies [26, 27].

There are several limitations of the present study that should be addressed. First, only contrast enhanced CT and/or MRI were used for the assessment of PVTT response, at 1, 3, and then every 3 months after SBRT. If liver vessel Doppler scan had been utilized in this study, the portal vein blood flow changes after SBRT might have been detected with higher frequency. Since Doppler exams could be repeated in shorter intervals, more information could have been collected in this respect. Second, the study was retrospective, with widely varied therapeutic modalities after SBRT for this group of patients, including TACE, surgical resection, and one liver transplantation. In addition, some patients received different number of TACE sessions. These could represent sources of bias for the observed data, and one should be cautious in the interpretation of the clinical outcomes. A well designed prospective study is warranted to validate the results.

## Conclusions

SBRT can be used as the first-line therapy for HCC patients with extensive PVTT originally considered unsuitable for surgical resection or TACE. The dynamic change of tumor thrombosis in time after SBRT has been described. Thrombus shrinkage and portal vein flow restoration can be achieved in the majority of cases. Thereafter, SBRT may enable patients to pursue consolidative local treatments, such as surgery and TACE procedure. Such therapeutic strategy may result in improved survival benefit, especially for those who do receive further therapies after SBRT.

## Abbreviations

3D-CRT: Three-dimensional conformal radiotherapy
AFP: Alpha-fetoprotein
CTCAE: Common Terminology Criteria for Adverse Events
D1cc: Maximal dose to 1 cc
HCC: Hepatocellular carcinoma
LC: Local control
mRECIST: Modified Response Evaluation Criteria in Solid Tumors
OARs: Organs at risk
OS: Overall survival
PD: Progressive disease
PR: Partial response
PVTT: Portal vein tumor thrombus
SBRT: Stereotactic body radiotherapy
SD: Stable disease
TACE: Transarterial chemoembolization
